# mSphere Legacy: A career built on suppressors

**DOI:** 10.1128/msphere.00568-26

**Published:** 2026-09-22

**Authors:** Thomas J. Silhavy

**Affiliations:** 1 Department of Molecular Biology, Princeton University 200547, Princeton, New Jersey, USA; Virginia-Maryland College of Veterinary Medicine, Blacksburg, Virginia, USA

**Keywords:** protein secretion, OM biogenesis, two-component systems

## Abstract

My career in research began in the early 1970s and is now ending. In the early years, I learned much from three wonderful mentors. My research has focused on *Escherichia coli*, and my studies were motivated by two basic questions: how does this bacterium build its complex cell envelope, and how does it regulate gene expression in response to external stimuli? Much of my success stemmed from searches for suppressors. None of what I write would have been possible without the graduate students and postdocs I have had the pleasure of supervising, and I apologize in advance for not highlighting the contributions of each of you. I have also profited enormously from the contributions of wonderful colleagues and of several valued collaborators, each brought new insights and indispensable expertise.

## COMMENTARY

I grew up in a small town, Wauseon, in the northwest corner of Ohio. Although I enjoyed science and math classes in high school, a career in research and academia never crossed my mind. I worked for several years in a local pharmacy, and so when it came time to go to college, I decided to go to pharmacy school at Ferris State University in Michigan.

At Ferris, I was captivated with organic chemistry and became an assistant to my first mentor, my medicinal chemistry professor George N. Holcomb. George had enormous influence on me. He opened my eyes to the possibility of a career in academics and research, and he convinced me to go to graduate school. Initially, I wanted to study organic chemistry, but George reasoned that there would be more opportunities in biochemistry and especially membrane biochemistry. I applied to several Big 10 schools, but on a bet, George persuaded me to also apply to schools on both coasts. The West Coast was a bust for me, but my acceptance at Harvard cost me a bottle of scotch.

I had a very lucrative offer to stay in Michigan as a pharmacist. Instead, I took a very significant pay cut and went to Boston. At the time, I knew I would regret it if I turned down the chance to go to Harvard; I had to give it a shot. Also, I knew that I could always return to pharmacy if I flunked out! Thankfully, my wife agreed.

## GRADUATE SCHOOL, HARVARD MEDICAL SCHOOL, 1971–1975

In the summer of 1971, I showed up at the Department of Biological Chemistry at Harvard Medical School. My first year in grad school was extremely challenging; a pharmacy education is perhaps not the best preparation for biochemistry, but I had several very helpful classmates. Initially, I wanted to work with Gene Kennedy, but he convinced me to join the lab of Winfried Boos instead. Gene wound up being the chair of my thesis committee and was helpful to me in many ways before and after graduation. Unlike Gene’s large lab, Winfried’s lab was small, so I got an abundance of hands-on guidance from him and we became very close.

As a graduate student with Winfried, I studied several different sugar transport systems in *Escherichia coli* using a variety of biochemical techniques. These studies focused on transport systems that have soluble periplasmic-binding proteins. The knowledge I gained from studying these non-cytoplasmic proteins provided a solid framework for my future studies of protein secretion and targeting described below.

Just prior to my final year in graduate school, Winfried accepted a professorship at the University of Konstanz in Germany. This meant that his planned year-long sabbatical at the Institute Pasteur would be cut to 6 months and then he would go to his new position. It also meant that I would have to go with him. Gene was especially helpful preparing for this trip. In fact, he arranged for me to be awarded a master’s degree, which allowed me to teach, and thus be paid, in Germany. This is why my A.M. and Ph.D. degrees were awarded in adjacent years.

At Pasteur, we joined the lab of Maxime Schwartz, and this turned out to be the beginning of a long and productive collaboration between Maxime and I. During this time, I learned about the maltose transport system (a founding member of the ABC superfamily of transporters), which contains components in each of the cellular compartments of *E. coli*, cytoplasm, inner membrane (IM), periplasm, and outer membrane (OM). My most important contribution to this transport system during this period concerned the OM protein (OMP) LamB (a.k.a. bacteriophage λ receptor). We were the first to propose that an OMP functions as a diffusion channel (1). Today, we understand that LamB belongs to a large class of molecules termed porins, which reflects this function. Indeed, LamB is now called maltoporin because, unlike the general porins, OmpF and OmpC, LamB prefers maltose and maltodextrin substrates.

Going back a bit, during my second year in graduate school, I took Jon Beckwith’s course and fell in love with bacterial genetics. About a year later, I learned that Jon was interested in figuring out how periplasmic proteins got to the periplasm. So, before I left for Europe, I arranged to do a postdoc with Jon.

## POSTDOCTORAL TRAINING, HARVARD MEDICAL SCHOOL, 1975–1977

Malcolm Casadaban, a graduate student in Jon Beckwith’s lab, had developed a genetic technique for constructing gene fusions. As recombinant DNA technology was just being developed, Malcolm’s remarkable technique used the bacteriophages λ and Mu instead. As a postdoc with Jon, I exploited Malcolm’s strategy to pioneer the use of reporter gene fusions for analyzing protein secretion and targeting. Indeed, we were the first to use gene fusions to address problems of cell biology (2). This technique is now widely used in all organisms but with fluorescent proteins instead of LacZ. The idea of using *lacZ* gene fusions was Jon’s, of course. However, it was my idea to use proteins of the maltose transport system as targets. This system provided numerous advantages, and the IM protein MalF, the periplasmic protein MalE, and the OMP LamB became paradigms for studying targeting to each of these cellular locations.

During this period, we realized that *lacZ* fusions to these *mal* genes conferred novel and unexpected phenotypes. As it turns out, exploitation of these phenotypes allowed genetic dissection of the signal sequence and identification of the genes encoding the secretion machinery and factors that catalyze protein folding in the periplasm of *E. coli*. Despite the usefulness of the fusion phenotypes, we did not completely understand their molecular basis until years later (3). Basically, if the hybrid LacZ protein is secreted either by a post-translational (SecB-dependent) pathway or a co-translational (signal recognition particle-dependent [SRP]) pathway, high-level production is lethal, but for different reasons. Folding of the LacZ component in the cytoplasm jams the secretion apparatus when the fusion is secreted by the SecB pathway, and jamming causes degradation of the essential SecY (jammed) complex by the FtsH protease (4). LacZ cannot fold in the cytoplasm, and so it does not jam the SecY complex when the fusion is secreted co-translationally by the SRP pathway. Rather, DsbA-dependent misfolding of LacZ produces a disulfide-bonded aggregate in the periplasm that is toxic (cysteines in LacZ do not form disulfide bonds in the reducing cytoplasmic compartment). Furthermore, low-level expression of hybrid LacZ proteins allows secretion to the periplasm at non-toxic levels by either secretion mechanism (3). Thus, as described below, LamB-LacZ and MalE-LacZ strains are inducer sensitive, and selection for inducer resistance and Lac^+^ yields signal sequence mutants. Cells producing low levels of each hybrid protein have little LacZ activity because most of it is secreted and cannot fold properly in the oxidizing periplasm. Selections for Lac^up^ yields mutations that compromise the secretion machinery, allowing more of the hybrid protein to accumulate folded LacZ in the cytoplasm. A secondary screen for temperature sensitivity allowed identification of essential genes required for protein translocation across the IM.

## INSTRUCTOR, HARVARD MEDICAL SCHOOL, 1977–1979

My first independent lab was established with two exceptional graduate students, Scott Emr and Mike Hall, and a technician. Scott and Mike set my lab on solid footing right from the start in two different research fields.

Scott and I were the first to identify and characterize mutations that alter the signal sequence using the inducer sensitivity of LamB-LacZ fusions (5). This work provided proof for the signal hypothesis of Gunther Blobel and Bernhart Dobberstein (6).

Mike and I initiated a project using *lacZ* operon fusions to analyze the complex regulatory mechanism that controls the reciprocal expression of the major OM porin genes *ompF* and *ompC* in response to environmental changes such as osmolarity. This strategy enabled us to distinguish regulatory genes from structural genes, a conundrum that had plagued the field previously (7).

Together with Howard Shuman (a graduate student in Jon’s lab), we also devised a general method using LacZ hybrid proteins to make antibodies directed against unknown or undetectable gene products using MalF as an example (8). Variations of this method using hybrid proteins containing sequences of MalE (a.k.a. MBP), GST, or epitopes are now available in kit forms from various manufacturers.

## SECTION HEAD/LAB DIRECTOR, FREDERICK CANCER RESEARCH CENTER, 1979–1984

Scott and I pioneered the use of suppressors of signal sequence mutations to identify components of the protein secretion machinery, proving that these suppressors restore export of the mutant precursor from the cytoplasm. Using this method, we identified the first gene, which we termed *prlA*, that specifies a component of the protein secretion machinery and showed that this gene resides in a large operon that encodes mostly ribosomal proteins (9). Later that same year, Don Oliver and Jon Beckwith (10) exploited the Lac phenotype of *lacZ* fusions described above to identify the gene *secA*. Ito et al. (11) isolated a temperature-sensitive mutation that mapped to the same chromosomal location as *prlA* and named the gene *secY*. The *prl* mutations are dominant suppressors of signal sequence mutations; the *sec* mutations are recessive. As described below, both types of mutations can be isolated in the same gene, and this discovery provided strong evidence that these genes encoded components of the secretion machinery. This dual nomenclature distinction is still used today, and homologs of *prlA/secY* are found in all organisms from simple *Mycoplasma* through the *Archaea* to mammals, where it is called Sec61α. By contrast, SecA is present only in prokaryotes and chloroplasts.

Based on our genetic analyses, Mike and I demonstrated that the transcriptional regulation of the major OM porin proteins is controlled by two genes, which we termed *envZ* and *ompR*. We concluded that EnvZ is a membrane-bound sensor that transfers environmental information to the DNA-binding protein OmpR, which then activates expression of the appropriate gene (12). This model remains fundamentally correct today. Six years after this work of Mike’s and mine, Fred Ausubel published a minireview in *Cell* that extrapolated sequence similarities to propose “two-component regulatory systems,” each containing a sensor and a regulator (13). Despite the uninformative name, the term and the concept caught on. We now know that two-component regulatory systems are commonly employed by prokaryotes, including plant and animal pathogens, for environmental sensing. There are even a few examples in eukaryotes as highly evolved as yeast and plants.

## PRINCETON UNIVERSITY 1984–PRESENT

### Sec/Prl

Using MalE as a model system, the late Phil Bassford’s lab identified suppressors of signal sequence mutations (termed *prlD*) that map to *secA* (14). In my lab, Lisa Gansheroff (undergrad) and Joan Stader (postdoc) confirmed this result and identified yet another suppressor gene termed *prlG* (15). We showed that *prlG* was allelic with *secE*, a gene that had recently been identified in the Beckwith lab. Since Jon’s mutations were not in the coding sequence, but rather affected gene expression, our results greatly strengthened the case that *prlG/secE* is important for protein secretion. We now know that SecE is an essential component of the secretion machinery and that homologs are found in all organisms. In mammals, PrlG/SecE is called Sec61γ.

Graduate students Kris Bieker-Brady and Bob Osborne, postdocs Ann Flower and Chris Harris, and I devised several clever genetic strategies that exploit the properties of the *prl* suppressors, including suppressor-directed inactivation, Sec titration, synthetic lethality, and *in vivo* protein crosslinking, to probe the process of protein translocation. We identified interactions between specific domains of Sec (Prl) proteins during translocation. The most revealing of these interactions is between the first periplasmic loop of SecY and the second periplasmic loop of SecE. The *prl* alleles that alter amino acids in these two locations are a synthetic lethal pair (16). Replacing these residues with cysteine results in a DsbA-dependent dominant-lethal phenotype, demonstrating a direct physical interaction between these two amino acids even though they are in different proteins (17).

The direct interaction described in the previous paragraph was illuminating because in the first crystal structure of the SecY complex solved by van den Berg et al. (18), the two amino acids that exhibit synthetic interactions are more than 20 Å apart. The solved structure represents the closed SecY complex, and our disulfide-bonded complex provided important clues about how the channel might open. Indeed, the dominant-lethal disulfide bond locks this channel into the open state, explaining why it is lethal. Based on this and other studies, we concluded that *prl* mutations either destabilize the closed complex or stabilize the open complex, and by doing so, they bypass the need for a signal sequence. SecB recognizes these signal-less proteins and directs them to SecA, which then engages the SecY complex (19).

### OmpR/EnvZ

The 1980s and 1990s were exciting times at Princeton for two-component signal transduction studies. Jeff and Ann Stock and Stan Leibler were studying their role in chemotaxis in *E. coli*. Austin Newton was studying their role in development in *Caulobacter crescentus*, and Bonnie Bassler was studying their role in quorum sensing in *Vibrio* species. During this period, Michele Igo (a postdoc) showed that the cytoplasmic domain of the membrane-bound sensor EnvZ has three biochemical activities: autokinase, OmpR kinase, and OmpR-P phosphatase (20). These results together with genetic studies by Jim Slauch, Frank Russo, Leslie Pratt (graduate students), and I led to the following model for the transcriptional regulation of the major OM porin genes by the EnvZ/OmpR two-component system. The important message here is that this two-component system does not work by simply turning the kinase on and off. It works by fine-tuning the level of OmpR-P.

The periplasmic domain of the sensor EnvZ monitors osmolarity, and conformational changes caused by changes in this environmental parameter alter the ratio of the kinase/phosphatase activities of the cytoplasmic domain to, in turn, vary the intracellular concentrations of the phosphorylated form of the response regulator OmpR. When medium osmolarity is low, the amount of OmpR-P is low. Under these conditions, OmpR-P binds to high-affinity sites at the *ompF* promoter to activate transcription through specific contacts between the α-loop region of OmpR and the carboxy-terminal domain of the α subunit of RNA polymerase. When media osmolarity is high, the amount of OmpR-P increases, allowing it to additionally bind low-affinity sites at *ompC* to activate its transcription and low-affinity sites at *ompF* to repress transcription by a mechanism that involves DNA looping (for review, see reference 21). This model has withstood the test of time. However, the actual inducing stimulus remains mysterious.

### RpoS

Leslie Pratt and I discovered that expression of *ompF* is specifically repressed in stationary phase by a mechanism that does not involve EnvZ/OmpR. Rather, this mechanism employs the stationary phase σ factor RpoS. Moreover, we identified a gene, *sprE* (a.k.a. *rssB*), which controls the levels of RpoS by targeting this σ factor to the ClpX/ClpP proteasome (22). SprE belongs to the response regulator family of two-component systems and thus represents the first such molecule that is involved in regulating protein stability. Surprisingly, Celeste Peterson (graduate student) showed there is no cognate kinase. RpoS degradation is controlled directly by ATP levels; degradation stops upon carbon starvation because ATP levels drop (23).

### CpxA/CpxR

Christine Cosma, Bill Snyder, and Paul Danese (graduate students) and I searched for suppressors of a variety of different envelope toxicities, including LacZ jamming of the Sec machine and the periplasmic toxicity of misfolded LacZ described above, to identify genes that specify periplasmic factors that combat protein-folding defects. These selections yielded mutations that constitutively activate another two-component regulatory system, CpxA/CpxR. Work from the lab showed that the sensor kinase/phosphatase, CpxA, senses misfolded proteins in the cell envelope and communicates this information by varying the intracellular levels of CpxR-P (by a mechanism analogous to that described above for EnvZ/OmpR). CpxR-P, in turn, controls expression of genes including *degP* (encoding a periplasmic heat-shock protease) and *dsbA*. We also obtained evidence that the extra-cytoplasmic heat shock σ factor σ^E^ (first identified by Carol Gross) also responds to misfolded proteins and controls expression of many genes involved in periplasmic physiology, some of which overlap with the Cpx regulon. The number of Cpx-inducing signals is very large, as is the regulon, but recent work suggests that its primary role is to respond to perturbations at the IM (for review, see reference 24).

We discovered two other envelope proteins that regulate the Cpx system. The gene for the OM lipoprotein NlpE was discovered using a multi-copy version of one of the envelope toxicity screens described above. Subsequently, Karen Otto (a postdoc) discovered that NlpE functions in surface sensing (25). Marcin Grabowicz (a postdoc) also discovered a role for NlpE in monitoring lipoprotein trafficking to protect cells from mislocalized lipoproteins in the IM (26).

Tracy Raivio (postdoc) showed that Cpx is positively autoregulated and subject to feedback inhibition (27). The *cpxP* gene lies next to the genes for *cpxRA* and is strongly induced by Cpx activation. CpxP inhibits Cpx by binding to the periplasmic sensing domain of CpxA. It also plays a role in mitigating the toxicity of misfolded envelope proteins (for review, see reference 24). Dan Isaac (a postdoc) showed that CpxP functions as an adaptor protein that directs misfolded proteins to the periplasmic DegP protease. Since there is no energy in the periplasm to remove the adaptor, CpxP is degraded along with its substrate, and this, of course, relieves CpxA inhibition (28). Another sacrificial periplasmic adaptor protein is described below.

### Outer membrane biogenesis

The OM contains four major components: lipoproteins; OMPs, which are transmembrane proteins that assume a β-barrel conformation; glycerophospholipids (PLs); and lipopolysaccharides (LPS). Each of these components is made in the cytoplasm or the IM, and they must be transported across the aqueous periplasm and assembled into a membrane on the other side of the peptidoglycan cell wall. Since all of this happens in an environment that lacks an obvious energy source, it poses an intriguing assembly problem that has fascinated me since graduate school.

#### Lipoproteins

An important first step in this endeavor was made by Hajime Tokuda with his discovery of the Lol pathway for lipoproteins. These proteins are translocated from the cytoplasm by the Sec machinery, and then lipids are added to what will become the amino terminal cysteine residue. The now lipidated protein is extracted from the IM by an ABC transporter (LolCDE) and handed off to a periplasmic chaperone (LolA) for transport across the periplasm to an OM assembly factor (LolB; for review, see reference 29). This finding suggested that other OM components may use periplasmic chaperones as well, and as will become apparent, ABC transporters play major roles in OM biogenesis.

#### LPS

The periplasm was known to contain proteins, such as SurA and Skp, which could function as chaperones for OMPs. An important initial observation was that null mutations in *surA* and *skp* have synthetic phenotypes that result in greatly reduced levels of several OMPs, suggesting that SurA and Skp are important redundant chaperones (30). This finding was also revealing for several other reasons. First, both genes are located in an operon downstream of the gene for a large OMP. The *skp* gene was downstream of *yaeT* (now called *bamA*), and *surA* was downstream of *imp* (now called *lptD*). Second, both operons were regulated by σ^E^, and there were mutations in *lptD*, described below, that drastically disrupted the OM.

Barbara Sampson (undergrad), Rajeev Misra (postdoc), and Spenser Benson (postdoc and then research scientist) devised a selection to identify mutations that altered the OM permeability barrier. As noted above, LamB, the OM maltoporin, is required for growth on maltodextrins. They selected for mutations that bypassed LamB by allowing growth on this carbon source. One of the mutations they identified is *lptD4213*, which profoundly disrupts the OM permeability barrier (31). Although the *lptD4213* strain was often used to facilitate the search for antibacterial compounds, the gene remained largely uncharacterized for over a decade.

Martin Braun (postdoc) built an *E. coli* strain in which LptD could be depleted and showed that the protein was essential. Upon depletion, the OM became denser, suggesting defects in lipid trafficking. However, with depletion strains, it is difficult to tell primary from secondary defects, so we cautiously concluded that LptD was required for OM biogenesis (32). A role in LPS transport was demonstrated by the Tommassen lab, who showed that in *Neisseria meningitidis*, where LPS is not essential, mutants lacking LptD made little LPS, and it was not on the surface (33).

At this point, I must digress to describe a very productive, long-time collaboration with Dan Kahne and Natividad (Natacha) Ruiz. Dan was a chemistry faculty member here for 16 years before moving to Harvard in 2004. Natacha began as a postdoc in my lab but then remained at Princeton as a research scientist before becoming a faculty member at Ohio State in 2010. I’ll describe how the collaboration started in the next section, but from here on, nearly all the work on the OM done in my lab was performed together with Dan and Natacha.

Using an epitope-tagged LptD, affinity purification, and depletion strains, we discovered that LptD exists in a complex with the essential lipoprotein now called LptE. Alessandra Polissi discovered two proteins, now called LptA and LptB, that are required for LPS transport, and her student Paola Sperandeo came to my lab to show that these proteins, together with a protein now called LptC, function in the same transport pathway. The final piece of the puzzle came from Natacha, who designed an elegant reductionist bioinformatic approach that took advantage of the small genome size of a gram-negative endosymbiont to discover two genes of unknown function whose homologs in *E. coli* turn out to be the missing components of an ABC transporter required for LPS transport (for review, see reference 34).

LPS is synthesized in the inner leaflet of the IM by the “Raetz pathway” and then flipped to the outer leaflet by the ABC transporter MsbA. Rather than use a periplasmic carrier like LolA, the seven Lpt proteins form a trans-envelope bridge with the LptBFGC ABC transporter in the IM connected to the LptDE complex in the OM by the periplasmic LptA. The ABC transporter pushes a continuous stream of LPS molecules across the bridge and directly onto the cell surface (The Pez Model, an appropriate name coined by Dan). This mechanism solves the energy problem by coupling ATP hydrolysis at the inner surface of the IM directly to the appearance of LPS molecules on the outer cell surface (35).

#### OMPs

For many years, I knew Dan Kahne was my colleague at Princeton, but we rarely interacted. That changed at a Banbury Conference entitled “Microbial Targets for Small Molecules” in late November 1999. At this conference, Dan talked about a derivative of vancomycin (vanc) he had synthesized, chlorobiphenyl vancomycin (CBPV), which killed vancomycin-resistant mutants of *E. coli*, presumably by a different but unknown mechanism than that used by vanc. After his talk, I convinced him to try to identify this unknown mechanism using genetics by isolating a mutant resistant to CBPV but not vanc, and so the collaboration began.

Upon returning to Princeton, I persuaded Natacha to join this collaboration, and she began teaching basic genetic techniques to several people, including Ulricke Eggert, in Dan’s lab. Since wild-type *E. coli* is naturally resistant to CBPV and vanc, we used strains carrying the *lptD4213* mutation to disrupt the OM permeability barrier. Using this strain, we discovered three mutations, all of which prevented expression of *yfgL* (now called *bamB*), a gene that specifies an OM lipoprotein that confers resistance to CBPV but not vanc. Because the lack of BamB also conferred resistance to moenomycin, but not to other antibiotics, we thought that BamB might somehow sense the status of the peptidoglycan (36).

I remember clearly the day Natacha came into my office telling me that the *bamB* mutations suppressed the sensitivity of *lptD4213* strains to CBPV not by altering the drug target but, remarkably, by altering the disrupted permeability barrier caused by the *lptD4213* mutation since the *bamB* mutations also confer resistance to bile salts. We went on to show that toxic small molecules can be used in strains with permeability defects to create specific chemical conditions that demand very specific suppressors, a technique we called chemical conditionality (37).

Initially we suspected that BamB would interact with LptD. However, coimmunoprecipitation experiments using His-tagged BamB revealed that BamB exists in complex with three other proteins now called BamA, an OMP, and the lipoproteins BamC and BamD. We demonstrated that BamA was essential and that the complex functioned to assemble OMPs in the OM (38). Later we identified a fifth component of the BAM complex, the nonessential lipoprotein BamE (39). We showed that depletion of BamD confers the same phenotypes as depletion of BamA (40) and that BamD binds the β-signal located near the carboxy terminus of the substrate to trigger the conformational change that activates BamA for OMP assembly (41).

We used chemical conditionality to identify another suppressor of *lptD4213* that mapped to *bamA* (42), raising the obvious question of why mutations in the BAM complex that assembles OMPs suppress mutations in the complex that assembles LPS on the cell surface. We wondered if perhaps this finding reflected a regulatory mechanism that jointly controlled these assembly machines to maintain homeostasis. A more attractive mechanism was suggested by the study of mutations in the gene encoding lipoprotein LptE. *lptE* mutations could be suppressed by not only mutations in *lptD*, which specifies a directly interacting partner but also by mutations in *bamA*, providing strong evidence that the LptD/E complex is assembled by the BAM complex (43). This discovery suggested that the LptD4213 mutant protein is assembly defective, and mutations in the BAM complex suppress that defect. Indeed, Dan’s group showed biochemically that LptD4213 stalls on the BAM complex; only a small fraction is assembled at the OM with LptE (44).

For almost a decade, we worked with scientists at Merck, principally Scott Walker and Terry Roemer, searching for small molecules that would interfere with OMP assembly. We found two. One of these, batimastat, inhibits the intramembrane protease RseP, thereby blocking the σ^E^ response and causing a lethal accumulation of unfolded OMPs in the periplasm (45). The other, MRL-494, inhibits OMP assembly. Although the *bamAE470K* mutation confers resistance to the drug, the mechanism by which it inhibits OMP assembly is still not clear (46). Although neither compound is useful clinically, both have proven to be useful experimental tools, especially MRL-494.

Christine Hagan, a graduate student in the Kahne lab, reconstituted the BAM complex *in vitro* and showed that its assembly of an OMP substrate requires SurA but not an energy source (47). The folding reaction is driven by thermodynamics; folded OMPs are incredibly stable structures. Dan’s lab has studied the biochemical mechanism of OMP folding in detail, showing that the β-signal of the substrate is engaged by the amino terminal β-strand of BamA’s barrel and that folding proceeds in a carboxy-to-amino-terminal manner by the addition of a template strand to the previous strand, starting with the β-signal (see reference 48 and references therein).

Dante Ricci (graduate student) isolated a temperature-sensitive lethal mutation *bamAE373K* that disrupts the interaction between the BamAB and BamCDE subcomplexes (49). Betsy Hart (graduate student) discovered that the *bamAE470K* mutation, which confers resistance to MRL-494, suppresses *bamAE373K*, and she showed that it does so by bypassing the essential requirement for BamD in OMP assembly (50). Indeed, this mutation bypasses the need for all Bam lipoproteins, demonstrating that the catalytic activity of the BAM complex resides solely in BamA (51).

As noted above, Skp has a chaperone function that is important in the absence of SurA. However, its primary role is to prevent accumulation of off-pathway OMP assembly intermediates (52). Skp removes stalled substrates from the BAM complex and delivers them to the DegP protease where, like the sacrificial adaptor CpxP, it is degraded along with its substrate (53). The mutation that confers resistance to MRL-494 and that bypasses the requirement for BamD, *bamAE470K*, blocks this Skp-dependent quality control mechanism, and *bamD* mutations that prevent this quality control mechanism also confer resistance to MRL-494 (54). Thus, BamD binds the β-signal of unfolded OMP substrates to initiate the assembly process, and it also helps remove defective substrates from BamA. MRL-494 appears to disrupt the conformational changes in BamA that are facilitated by BamD.

Despite the seemingly discrete projects of protein secretion and OM biogenesis, which were started at the beginning and near the end of my career, respectively, I see striking commonalities between the SecYEG and BAM complexes. Just like the SecYEG complex, the BAM complex exists in closed and open forms. To maintain intact permeability barriers of both the IM and the OM, the cell must keep these complexes in a closed state until needed. Both complexes require a peptide signal that is present at one end of the substrate to trigger formation of the active open form, and both complexes allow lateral exit of the substrate from an interior channel or lumen into a membrane. I am also struck by the similarities between the suppressors of signal sequence mutations and the *bamAE470K* mutation. The *prl* mutations bypass the need for a signal sequence, and the *bamAE470K* mutation bypasses the need for BamD, the component of the BAM complex that recognizes the β-signal. Synthetic interactions between *prl* mutations lock the SecYEG complex in an open lethal state (17), and the *bamAE470K* mutation confers dominant permeability defects in the OM (54). I think both mutations bias complex equilibrium to the open, active form.

#### PLs

Cellular components that transport PLs to the OM have proven the most difficult to identify for reasons that will become obvious as this story unfolds.

Juliana Malinverni (postdoc) noticed that homologs of an ABC transporter found in *Arabidopsis* that transports phosphatidic acid in chloroplasts exist in *E. coli* encoded in an operon that is now called *mlaBCDEF*. In other gram-negative bacteria, the gene now called *mlaA* is also interspersed with these genes; in *E. coli*, it is unlinked. MlaA is an OM lipoprotein, MlaC is in the periplasm, and the other Mla proteins constitute the IM ABC transporter. Juliana showed that mutants lacking any one of these proteins are sensitive to SDS-EDTA. To gain insight into the function of the system, she exploited that phenotype to isolate suppressors. All 51 of her suppressors increase expression of the gene encoding the enzyme PldA, which hydrolyzes PLs in the outer leaflet of the OM. Juliana concluded that the Mla system also functions to maintain OM lipid asymmetry, not by destroying PLs but, rather, by transporting them back to the IM (55).

Bert van den Berg’s lab solved the structure of MlaA showing that the protein forms a doughnut that is embedded in the OM’s inner leaflet with a central amphipathic pore. PLs from the outer leaflet diffuse through the pore where they are captured by MlaC, which binds PLs with high affinity. MlaC then shuttles to the IM where it is unloaded by the ABC transporter leaving PLs in the IM (56).

Here, I must again diverge to introduce another close collaborator, KC Huang. I got to know KC well when he was a postdoc at Princeton in the mid-2000s with my colleague Ned Wingreen. When I need help with single-cell or biophysical methods, I turn to KC, and much of the work that follows would not have been possible without him.

Holly Sutterlin (graduate student) isolated a dominant mutation in *mlaA* that we called mlaA* (57). The van den Berg lab showed that this mutation disrupts one of the helices that form the doughnut structure, allowing PLs from the inner leaflet to flow backward into the outer leaflet (56). Kerry May (postdoc) showed that the fatty acids released by PldA stimulate production of LPS (58). This excess LPS destabilizes the OM causing vesiculation in mlaA* cells. If cells are growing, this is not a serious problem. However, when they starve, OM lost by vesiculation is replaced by lipid flow from the IM to the OM that continues until the increased turgor pressure of the cytoplasm ruptures the IM even in the presence of energy poisons. This discovery demonstrates the existence of a high flux energy-independent intermembrane PL transporter (57).

To identify proteins involved in this high-flux flow, Jackie Grimm (graduate student) screened transposon insertion libraries for mutations that slow lipid flow. She discovered that mutations in *yhdP* slowed lipid flow by 50%, delaying lysis, but as a result, the weakened OM ruptured before the IM (59).

When we first viewed the model structure of YhdP predicted by AlphaFold, we knew immediately that this protein is a PL transporter. The structure is a long tube with a hydrophobic groove that closely resembles the hydrophobic groove formed by the Lpt proteins that form the bridge for LPS transport. This groove shields the hydrophobic acyl chains of the PLs as they cross the aqueous periplasm.

YhdP is an AsmA-like protein, and there are six of them in *E. coli*. Natacha’s lab and the lab of Stephen Trent showed that any one of three can support life, YhdP, TamB, and YdbH (60, 61). Unlike YhdP, TamB and YdbH have interacting partners necessary to form the intermembrane bridge. This redundancy is what made finding these transporters so difficult.

#### OM structure and function

Recently, there have been fundamental changes in our views of both OM structure and its function. Hiroshi Nikaido discovered that the OM is an asymmetric bilayer shortly after I started my postdoc. As he notes in his influential review, that “paper was rejected twice because a reviewer insisted that asymmetry was thermodynamically impossible” (62). The saturated acyl chains of LPS and the tight lateral interactions between LPS molecules mediated by divalent cations such as Mg^2+^ resulted in a gel-like structure that explained the major function of the OM as a permeability barrier. Influenced heavily by the fluid mosaic model of Singer and Nicolson (63), thus, it was generally thought that the surface of *E. coli* had randomly dispersed OMPs floating in a sea of LPS.

Georgina Benn, as a graduate student in the lab of Bart Hoogenboom, devised a way to look at the surface of live *E. coli* using atomic force microscopy, and this work overturned the standard view of OM structure. The OM surface is not a sea of LPS, rather it is covered with a protein lattice. The lattice is made up of pore-like structures in imperfect hexagonal symmetry (64). The pore-like structures are the porins, and the hexagons are imperfect because they also contain other OMPs. There are small patches of phase-separated LPS, but LPS also serves to fuse the lattice together (65). If you think of the hexagons as tiles, then LPS is the grout.

As a postdoc in my lab, Georgina went on to show that OmpA, which binds to the peptidoglycan, acts as a fence post to immobilize the OMP lattice. This coupling of two cellular structures produces a strong composite that contributes substantially to OM mechanical strength. As an analogy, think of rebar in concrete, a common composite. It also explains why mutants lacking OmpA have pleiotropic phenotypes (66).

These new atomic-level findings, along with other results, have driven another major change: the realization that the OM contributes significant mechanical strength to the gram-negative cell, which makes sense given that the peptidoglycan layer is probably only one molecule thick. This view was first suggested by work showing that phage λ encodes proteins needed to lyse the OM (67). Recent developments in microfluidics, microscopy, and other computational and biophysical methods have provided direct evidence for this mechanical role (for review, see reference 68).

## FINAL THOUGHTS

Over my career, I have been at a medical school, a research institution, and a university. Each of these organizations has advantages and disadvantages, but I have to say that I like the university the best. Here, I am constantly surrounded by smart, enthusiastic young people who are trying hard to find their place in the world. The only person that gets older is me, as most of the people surrounding me stay the same age forever. Yes, I must teach, but it has never been overwhelming for me; I love to teach. You haven’t really mastered a subject until you teach it, and it is rewarding to see the look in students’ eyes the exact moment when confusion turns to clarity.

Academia is a wonderful career. On the wall in my office is a picture of all the people who ever worked in my lab (Table 1). They live all over the world, and I remain in touch with nearly all of them, and many have become close friends. We have a lab reunion about every 5 years, and it is an event that many alumni look forward to. It is such a pleasure to see how each of them grew into the professionals that they now are. There is also the unparalleled joy of discovering something truly new and the privilege of traveling the world, telling people about it. I know people in many countries well enough to have dinner with them at their homes.

**TABLE 1:** Lab alumni

Graduate students	Postdocs	Visiting scientists	Lab technicians	Assistants
Neca Algood	Georgina Benn	Michael Berman	Susan Hanley-Way	Susan DiRenzo
Kristina Bieker-Brady	Spencer Benson	Andy Binns	Dolores Jackson	Sylvia Lucas
Valerie Carabetta	Martin Braun	Erhard Bremer	Vanessa Register	Jennifer Munko
John Carlson	Irina Mikheyeva-Bridges	Tom Ferenci	Brenda Shaffer	
Patricia (DiGiusepe) Champion	Christina Bowers	Neils Fiil	Liya Shi	
Gitanjali Chimalakonda	Charles Cowles	Lila Gierasch		
Ashton Combs	David Dolinger	Nina Hoyland-Kroghsbo		
Christine Cosma	Ann Flower	Steen Pedersen		
Paul Danese	Marcin Grabowicz	Manjula Reddy		
Amy Duguay	Randi Guest	Abigail Salyers		
Matthew Dufner (Masters)	Chris R. Harris	Joan Slonczewski		
Robert Dwyer	Anne Hiniker	Friederike Turnowsky		
Scott Emr	Weihong Hsing	George Weinstock		
Steve Garrett (JHU)	Michele Igo	Richard Wolf		
Katherine Gibson	Daniel Isaac	Ryland Young		
Jackie Grimm	Linda Kenney			
Mike Hall	Diane Kiino			
Nicholas Hand	Anna Konovalova			
Jill Harper	Juliana Malinverni			
Betsy Hart	Zachary Maschmann			
Janet Huie	Kerrie May			
Cassandra Korsvik	Rajeev Misra			
Fion Lau	Angela Mitchell			
Tara Mahoney	Aileen O’Connell			
Mark Mandel	Karen Otto			
Anne McCabe	Tapio Palva			
Andrew McCandlish	Craig Parker			
Faye Morris	Johan Paulsson			
Irene Ogievetskaya	Gregory J. Phillips			
Robert Osborne	Tracy Raivio			
Celeste Peterson	Beth Rasmussen			
Leslie Pratt	Natividad Ruiz			
Dante Ricci	John Shultz			
Frank Russo	Joan Stader			
Jaclyn Schwalm	Sha Tao			
Joseph Sklar	Alejandro Ureta			
Jim Slauch	Fred Warren			
Garner Soltes	Muhammed Ammar Zafar			
Paola Sperandeo				
William Snyder				
Holly Sutterlin				
Mark Taylor (masters)				
Ron Taylor (UMBC)				
Nancy Trun				
Johna Van Stelten				

However, I am well aware that establishing yourself in an academic career is certainly not easy. My best advice to young people who strive for this career is to pick something to study that you love with passion. Go to the best schools that you can, and when it is time to make a decision about postdoctoral work, go to the best lab. I was lucky to get into Harvard, but I think I made the most of that opportunity, and when I decided bacterial genetics was for me, I went to what I thought was the best lab in the world.
